# A *PiggyBac*-Based Recessive Screening Method to Identify Pluripotency Regulators

**DOI:** 10.1371/journal.pone.0018189

**Published:** 2011-04-18

**Authors:** Ge Guo, Yue Huang, Peter Humphreys, Xiaozhong Wang, Austin Smith

**Affiliations:** 1 Wellcome Trust Centre for Stem Cell Research, Department of Biochemistry, University of Cambridge, Cambridge, United Kingdom; 2 Wellcome Trust Sanger Institute, Hinxton, United Kingdom; 3 Department of Biochemistry, Molecular Biology and Cell Biology, Northwestern University, Evanston, Illinois, United States of America; Instituto de Medicina Molecular, Portugal

## Abstract

Phenotype driven genetic screens allow unbiased exploration of the genome to discover new biological regulators. Bloom syndrome gene (*Blm*) deficient embryonic stem (ES) cells provide an opportunity for recessive screening due to frequent loss of heterozygosity. We describe a strategy for isolating regulators of mammalian pluripotency based on conversion to homozygosity of *PiggyBac* gene trap insertions combined with stringent selection for differentiation resistance. From a screen of 2000 mutants we obtained a disruptive integration in the *Tcf3* gene. Homozygous *Tcf3* mutants showed impaired differentiation and enhanced self-renewal. This phenotype was reverted in a dosage sensitive manner by excision of one or both copies of the gene trap. These results provide new evidence confirming that *Tcf3* is a potent negative regulator of pluripotency and validate a forward screening methodology to identify modulators of pluripotent stem cell biology.

## Introduction

Genome-wide loss of function screening in the diploid mammalian genome is hindered by the requirement for homozygosity. Although RNA interference approaches have been applied, this only reduces rather than eliminates gene expression, currently lacks genome coverage in the mouse, and is subject to off-target effects. An alternative possibility is to exploit embryonic stem (ES) cells deficient for the Bloom syndrome tumour suppressor gene (*Blm*) [1], [2]. *Blm* encodes a RecQ helicase and mutant ES cells exhibit an elevated frequency of non-sister chromatid exchanges. Loss of heterozygosity (LOH) occurs at a rate of 4.2×10^−4^ per cell per locus per generation. This incidence predicts that on average a homozygous mutant should arise from a single heterozygous cell within 14 duplication cycles. A previous functional screen using *Blm*-deficient ES cells identified homozygous retroviral gene trap mutations in the DNA mismatch repair (MMR) pathway [2]. From 10,000 gene traps, multiple hits were identified in one gene, mismatch homolog 6 *(Msh6)*. This demonstrated the potential for homozygous screening for a selectable phenotype in ES cells, but also highlighted the insertion bias of retroviral mutagenesis.

*PiggyBac* (PB) transposition is highly efficient in human and mouse cells [3], [4]. Recently PB transposon based gene trap mutagenesis was applied in a new MMR screen in *Blm*-deficient ES cells [5]. Homozygous mutations in all four known MMR factors were recovered from 14,000 *PB* insertions, consistent with evidence that PB transposition has a broader spectrum of genome coverage than retroviral insertion.

Self-renewal of mouse ES cells is traditionally maintained by culture in serum using the cytokine leukaemia inhibitory factor (LIF) [6], [7]. Upon withdrawal of LIF, ES cells commit to differentiation under the influence of serum-factors or, in serum-free conditions, of autocrine fibroblast growth factor 4 (Fgf4) [8]. Disruptions in genes that mediate commitment or repress pluripotency circuitry are anticipated to reduce dependency on LIF. Here we used a PB transposon gene trap system in *Blm*-deficient ES cells to conduct a pilot screen for recessive mutations that could confer differentiation resistance.

## Results

**Figure 1:**
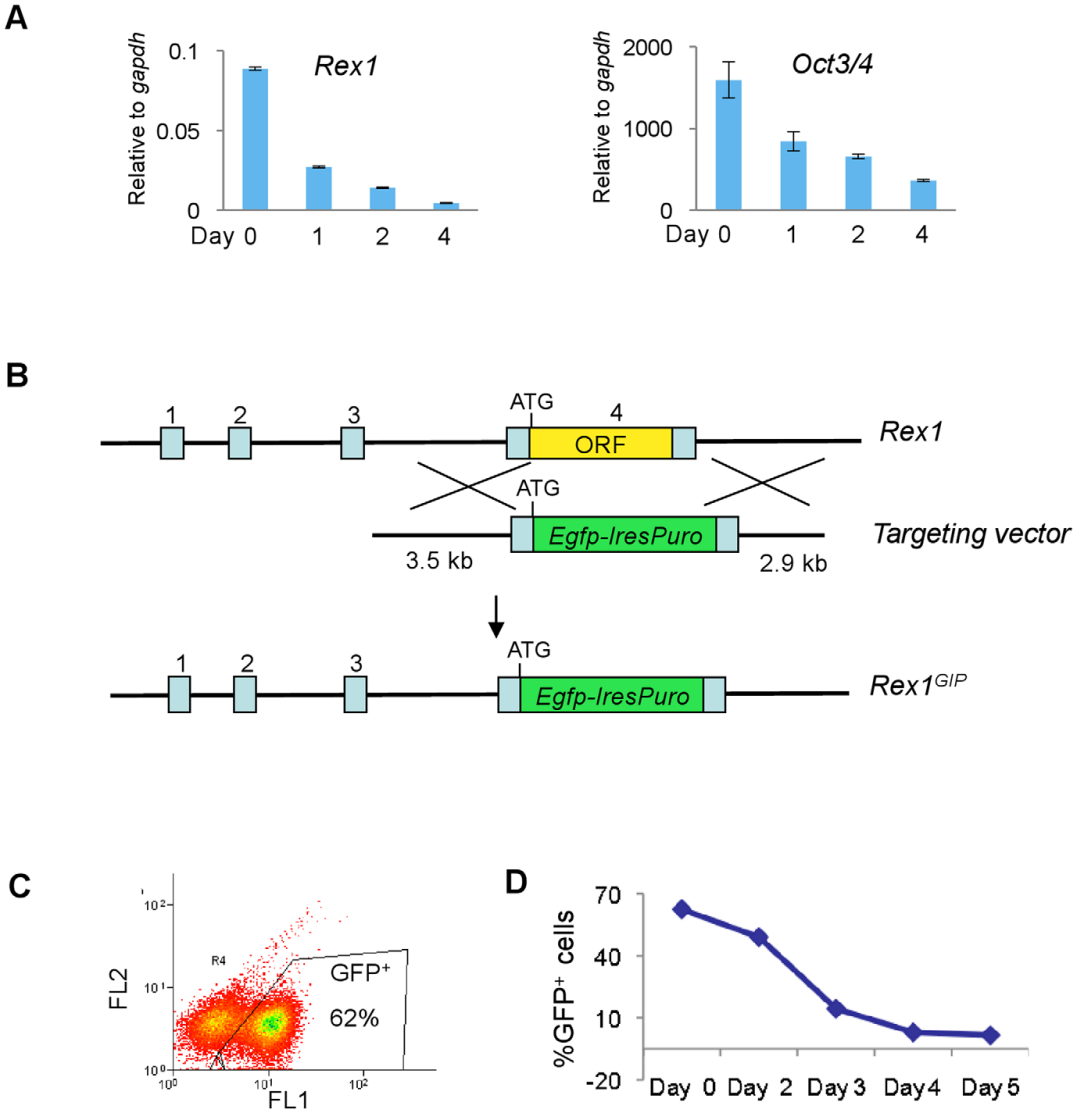
Generation of *Rex1* reporter cells. **A**. qRT-PCR analysis of *Rex1* and *Oct4* mRNA during monolayer differentiation in N2B27. **B**. Strategy to create the *Rex1^GIP^* knock in allele. **C**. Flow cytometry of a representative Rex1-Egfp profile in undifferentiated NN97-5 cells. E. Flow cytometry of Rex1-Egfp population in NN97-5 cells during monolayer differentiation in N2B27.

Implementing a recessive screen requires a strategy to identify and isolate rare phenotypes of interest. In the context of ES cell self-renewal, rapid and stringent selection is required because a fraction of cells invariably escape initial commitment. Such cells will subsequently expand under paracrine stimulation if differentiated cells are not eliminated [9], [10]. *Rex1* (*Zfp42*) is a specific marker of naïve undifferentiated ES cells [11]. It is down-regulated at the onset of differentiation more rapidly than the commonly used Oct4 marker (Fig. 1A). We therefore constructed a selectable *Blm*-deficient ES cell line by inserting *eGFPIresPuro* into the *Rex1* genomic locus via homologous recombination (Fig. 1B). The resulting NN97-5 cells expressed GFP in 60–80% of the population (Fig. 1C), consistent with the known mosaic expression of Rex1 in serum [11], [12]. Upon plating for differentiation, the proportion of GFP positive cells declined rapidly (Fig. 1D). By day 5, only 2–3% of cells remained GFP positive.

**Figure 2:**
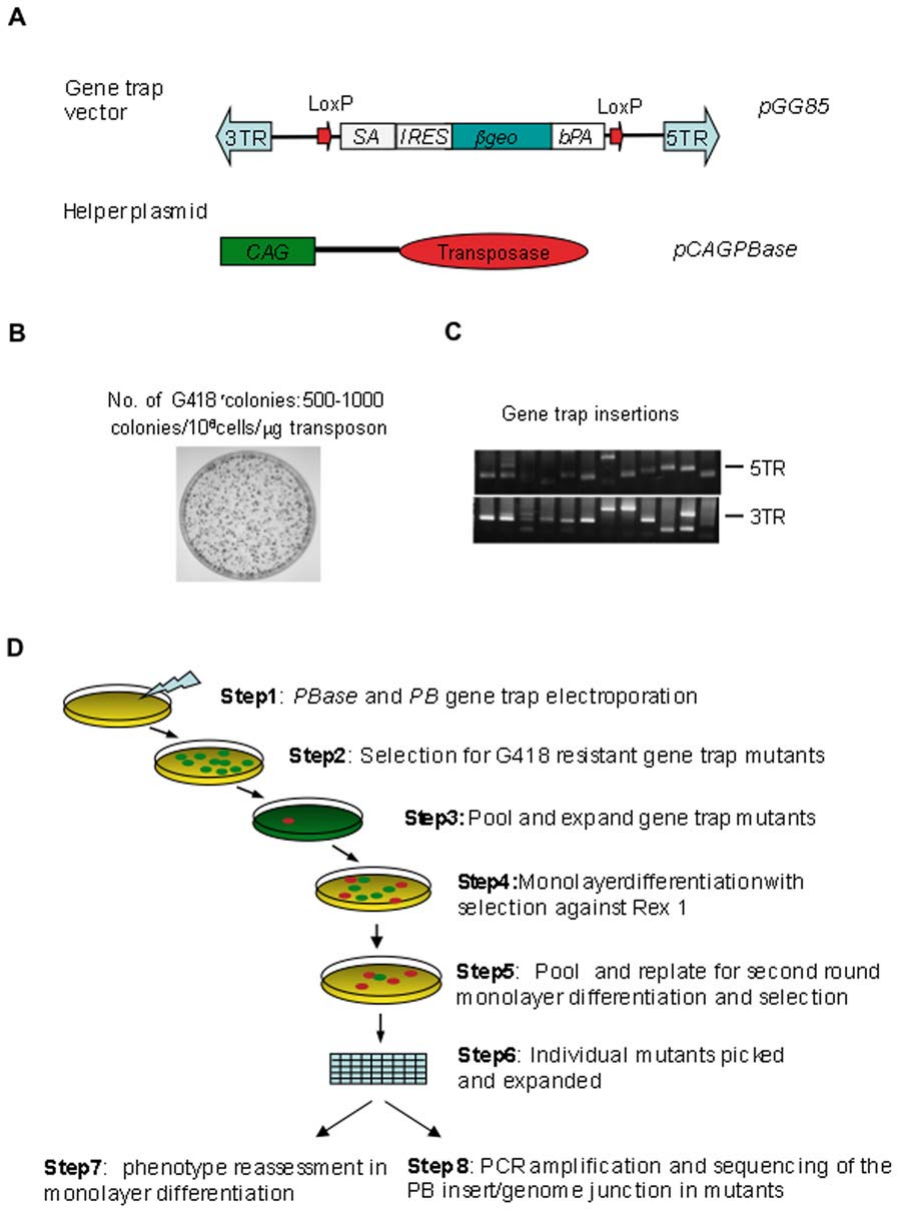
*piggyBac* mutagenesis and monolayer differentiation screen. **A**. Binary *piggyBac* gene trap system composed of gene trap vector, *pGG85*, and transposase expressing helper plasmid, p*CAGG-PBase*. **B**. G418 resistant colonies produced by co-electroporation of 1 µg of *pGG85* and 3 µg of helper plasmid. **C**. Splinkerette PCR amplified genome junction flanking PB insertions indicating the number of PB inserts in each clone. **D**. Schematic representation of monolayer differentiation screen.

We used a binary PB transposon delivery method for gene trap mutagenesis. This comprises a PB gene trap vector, *pGG85*, and a helper plasmid, *pCAGPBase* [4], that provides the transposase for vector/chromosome transposition (Fig. 2A). *pGG85* carries a promoter-less gene trap cassette, *SAIRESβgeo* [13]. The PB 5′ terminal repeat region (5*TR*) contains an RNA polymerase II promoter [14]. Therefore we positioned the *SAIRESβgeo* cassette in opposite orientation towards the 3′ terminal (*3TR*) (Fig. 2A). We included l*oxP* sites to enable reversion by Cre-mediated excision of the *SAIRESβgeo* cassette.

PBase mediated vector-chromosome transposition is very efficient. To restrict the number of integrations it is important to determined an appropriate ratio of transposase and transposon vector [5]. Electroporation of 2×10^6^ ES cells with 1 µg pGG85 and 3 µg pPBase yielded 500–1,000 G418 resistant colonies. Splinkerette PCR amplification [15] from 24 randomly picked clones indicated one or two PB insertions in most clones (Fig. 2B and 2C). We therefore employed this 1∶3 ratio.

The screening strategy is depicted schematically in Figure 2D. A pilot scale gene trap library was prepared by transfecting a total of 10^7^ NN97-5 cells in 5 electroporations as above. After twelve days under selection in G418, plates were harvested in two separate pools, each containing about one thousand clones and expanded for a further 48 hours. This period of 14 days since transfection is sufficient to allow for at least one homozygous conversion event at the majority of loci. Cells from each pool were then separately plated in N2B27 medium without serum and LIF. These conditions lead to neural differentiation of ES cells [16]. Untransfected NN97-5 cells were plated as a control. Five days later, puromycin was applied for two days to remove differentiating *Rex1* negative cells. LIF was added at the same time to maximize self-renewal of persisting undifferentiated cells. Recovered cells were replated for a second round of differentiation. Ten days later, over 100 undifferentiated colonies were evident in pool 1, while pool 2 and the NN97-5 control plates showed only around 10 colonies. Twenty colonies were picked from pool 1 for further analysis.

**Figure 3:**
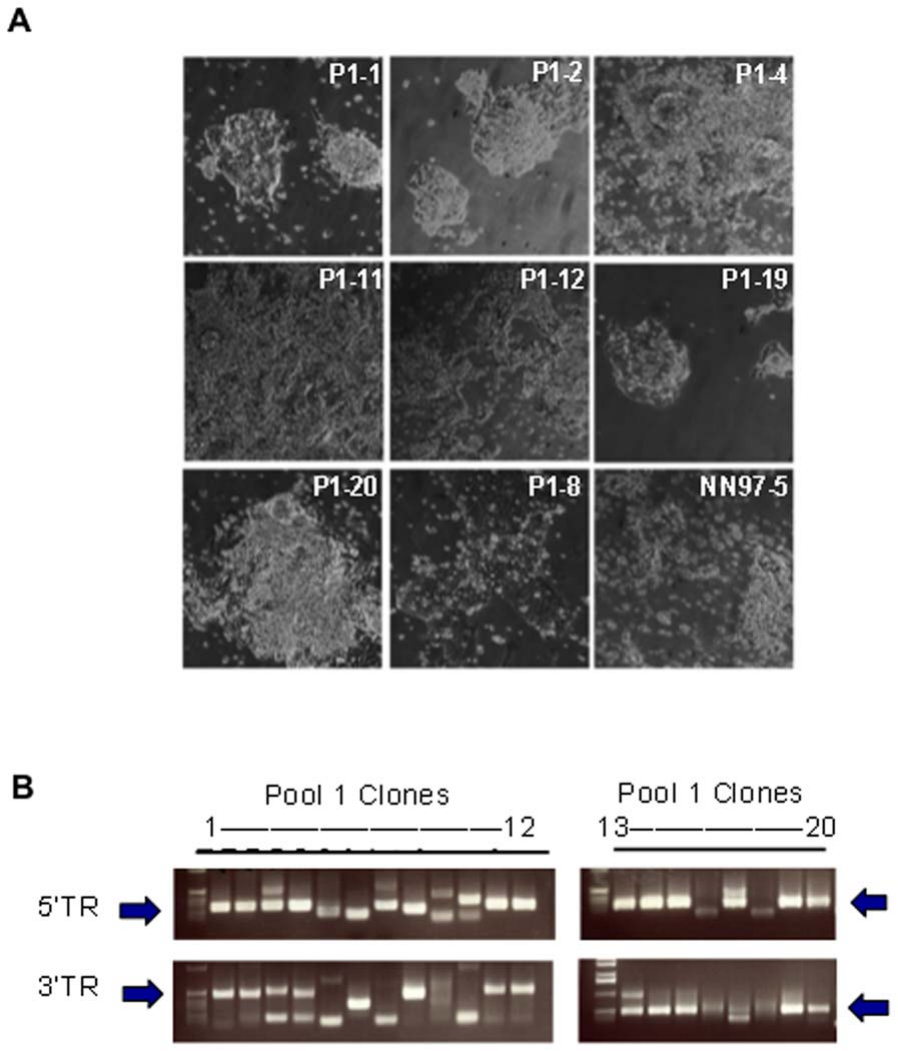
Gene trap mutants from monolayer differentiation screen. **A**. Images show typical differentiated and non-differentiated morphologies after 7 days monolayer neural differentiation assay. P1-1, P1-2, P1-4, P1-11, P1-12, P1-19 and P1-20 are clones carrying *Tcf3* gene trap mutation. **B**. Splinkerette-PCR amplified genome junctions flanking PB inserts. Gel images showing the genome junction flanking PB 5′ terminal repeat region (5′TR) and 3′ terminal repeat region (3′TR). Arrows indicate that a 500 bp 3′TR fragment and a 300 bp 5′TR fragment were amplified in multiple clones. Sequencing locates this band to *Tcf3* locus.

**Figure 4:**
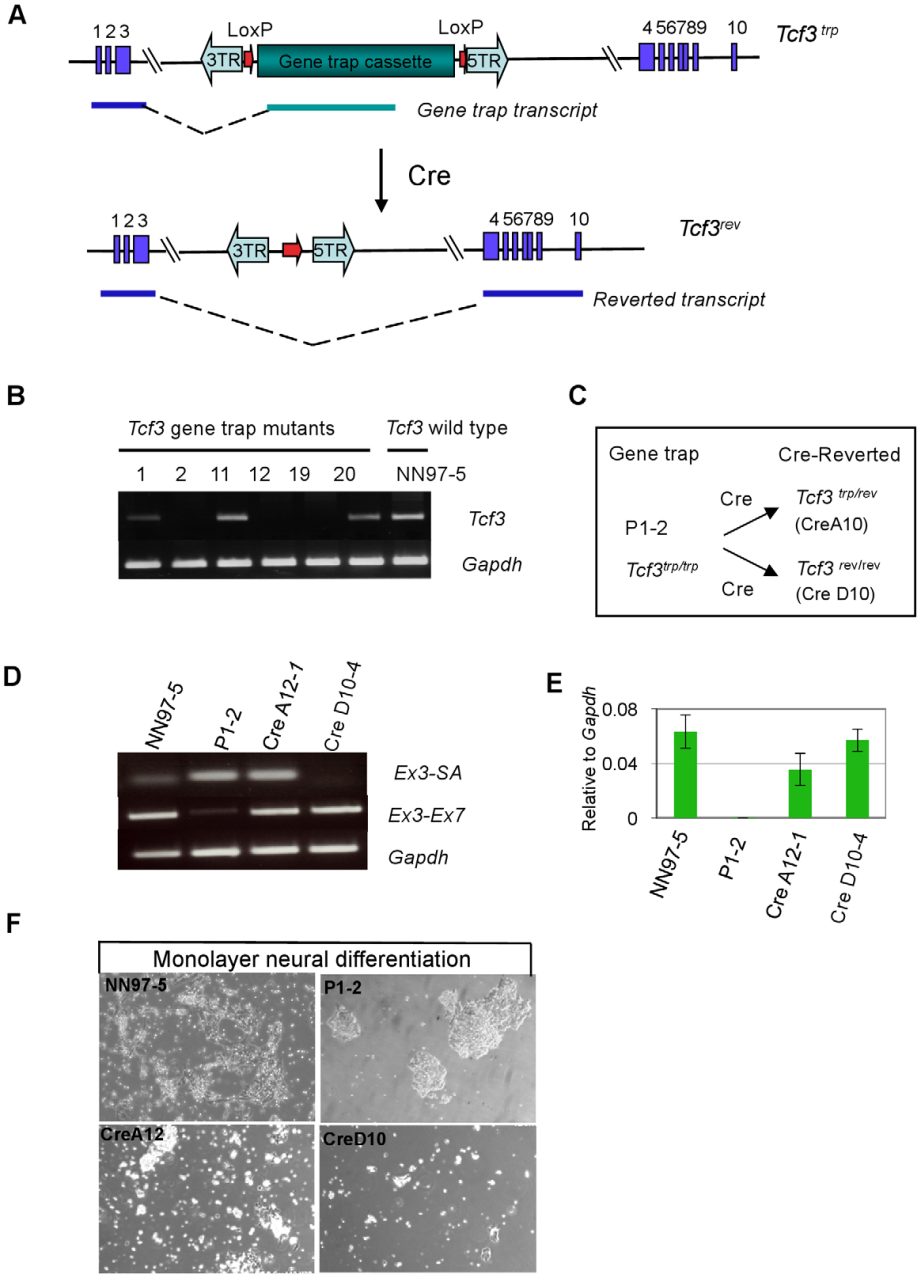
*Tcf3* gene trap mutants. **A**. *Tcf3* gene trap (*Tcf3^trp^*) and Cre-reverted *(Tcf3^rev^)* alleles. Cre recombination deletes the gene trap cassette to leave a reverted allele retaining the PB terminal repeats. **B**. RT-PCR analysis of *Tcf3* expression in gene trap mutants. *Tcf3* mRNA was not detected in clones P1-2, P1-12 and P1-19 but evident in clones P1-1, P1-11 and P1-20. **C**. Diagram showing generation of het or homozygous reverted cells. **D**. RT-PCR analysis of *Tcf3* gene trap (*Ex3-SA*) and *Tcf3* wild type (*Ex3-Ex7*) transcripts. CreA12-1 and CreD10-4 are subclones of CreA12 and CreD10. **E**. qRT-PCR analysis of *Tcf3* expression. F. After 9 days monolayer differentiation multiple ES cell colonies formed from *Tcf3* homozygote P1-2, but not from parental NN97-5 or revertant CreA12 or CreD10 cells.

Expanded clones were assessed for resistance to differentiation. Six clones produced mostly undifferentiated ES cells in monolayer neural differentiation conditions. The remainder showed high levels of differentiation (Fig. 3A and Table 1). We used splinkerette PCR amplification and sequence analysis to identify the insertion sites. All 6 carry the same PB integration in the third intron of the T-cell factor 3 (*Tcf3*) gene (Fig. 3B and 4A). This insertion was also identified in 4 of the differentiating clones (Table 1). We examined *Tcf3* expression by RT-PCR in Tcf3 mutants (Fig. 4B). *Tcf3* mRNA was undetectable in non-differentiating clones but present in the differentiating clones. This indicates that differentiating cultures with the *Tcf3* insertion might be heterozygous.

**Table 1:** Monolayer neural differentiation of individual gene trap clones.

Gene trap clones	Monolayer Differentiation	Gene trap clones	Monolayer Differentiation
P1-1*	D	P1-12*	Non D
P1-2*	Non D	P1-13*	Non D
P1-3	D	P1-14*	Non D
P1-4*	D	P1-15*	Non D
P1-5	D	P1-16	D
P1-6	D	P1-17	D
P1-7	D	P1-18	D
P1-8	D to flat cells	P1-19*	Non D
P1-9	D	P1-20*	D
P1-10	D	P2-1	D
P1-11*	D	P2-2	D

Monolayer neural differentiation of twenty clones from gene trap mutation pool 1 is presented. Clones with *Tcf3* mutation are labelled with “*”. Two clones from mutant pool 2 were also included as a control for monolayer differentiation assay. “D” represents clones showing extensive neural differentiation. “Non-D” represents cells showing predominantly undifferentiated ES cell morphology. P1-8 cells differentiated to flat non-neural cells.

To establish a causative link between the *Tcf3* mutation and differentiation deficiency, a homozygous *Tcf3* gene trap clone, P1-2, was transfected with a Cre expression plasmid. Cre recombination should remove the gene trap cassette and revert the induced mutation (Fig. 4C). Transfected cells were plated at low density for clonal expansion. By RT-PCR we identified clones that express wild type *Tcf3* mRNA (Fig. 4D). These included one clone, CreA12, which expressed both the gene trap transcript and the wild type *Tcf3* mRNA (Fig. 4D). Sub-cloning confirmed that CreA12 was not a mixed population but a clone in which only one *Tcf3* allele had been repaired. Consistent with heterozygosity, *Tcf3* transcript level in CreA12 cells was around 50% of that in parental NN97-5 cells (Fig. 4E). Whereas P1-2 cells formed abundant undifferentiated ES cell colonies in serum-free culture without LIF, homozygous repaired CreD10 cells rapidly differentiated (Fig. 4F). Heterozygous CreA12 cells initially formed a mixture of undifferentiated and differentiated cells, but by day 9 had mostly differentiated with few remaining ES cells. Phenotype reversion confirms that the *Tcf3* mutation is causal for enhanced self-renewal. Partial resistance to differentiation explains why heterozygous clones could be recovered in the screen and indicates dosage sensitive activity of Tcf3.

**Figure 5:**
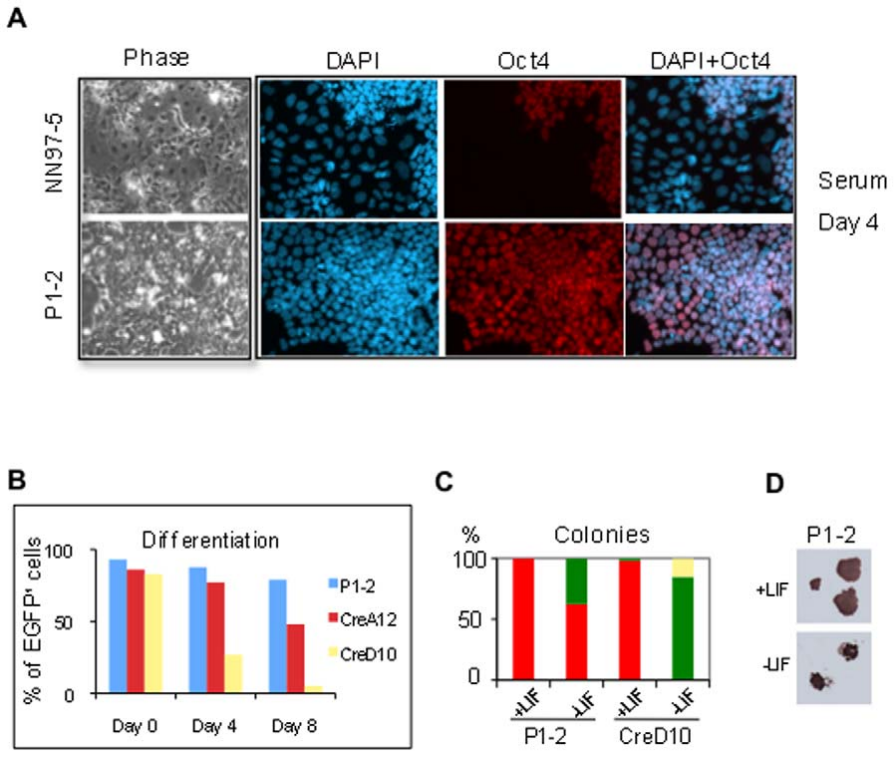
*Tcf3* deficiency suppresses serum-induced differentiation. **A**. Parental NN97-5 cells differentiate after 4 days in serum without LIF while *Tcf3* gene trap mutant P1-2 cells remain undifferentiated and retain uniform Oct4 expression in serum. **B**. Flow cytometry analysis for Rex1-EGFP positive cells during monolayer differentiation in serum. P1-2, Tcf3 gene trap mutant; CreA12, heterozygous Tcf3 Cre-revertant; CreD10, homozygous Tcf3 Cre-revertant. Graph shown is a representative of two independent experiments. **C**. *Tcf3* mutant (P1-2) and the *Tcf3* reverted cells were plated at single cell density in serum with or without LIF for colony forming assay. Colonies were stained after 9 days for alkaline phosphatase (AP) activity and colony numbers were quantified manually. Undifferentiated colonies are showing in red in figure and partially differentiated showing in green and differentiated showing in yellow. **D**. Images show typical AP positive morphologically undifferentiated ES cell colonies generated by P1-2 cells in serum with or without LIF. The experiment has been repeated once.

In the absence of LIF, serum induces heterogeneous non-neural differentiation of ES cells [10]. We tested P1-2 cells in these conditions and observed that a large fraction of cells retained undifferentiated ES cell morphology and Oct4 expression (Fig. 5A). They also maintained a high proportion of Rex1-GFP positive cells (Fig. 5B). In contrast, CreD10 cells showed rapid loss of GFP while *Tcf3* heterozygous CreA12 cells showed a more gradual reduction. We examined clonal propagation in the absence of LIF, a rigorous test of self-renewal efficiency. CreD10 cells produced only fully differentiated and mixed colonies (Fig. 5C). In contrast P1-2 cells formed entirely ES cell containing colonies. These colonies showed more differentiation than in the presence of LIF, however, and were smaller (Fig. 5D). Thus *Tcf3* deletion confers heightened resistance to differentiation in serum but does not substitute fully for LIF.

**Figure 6:**
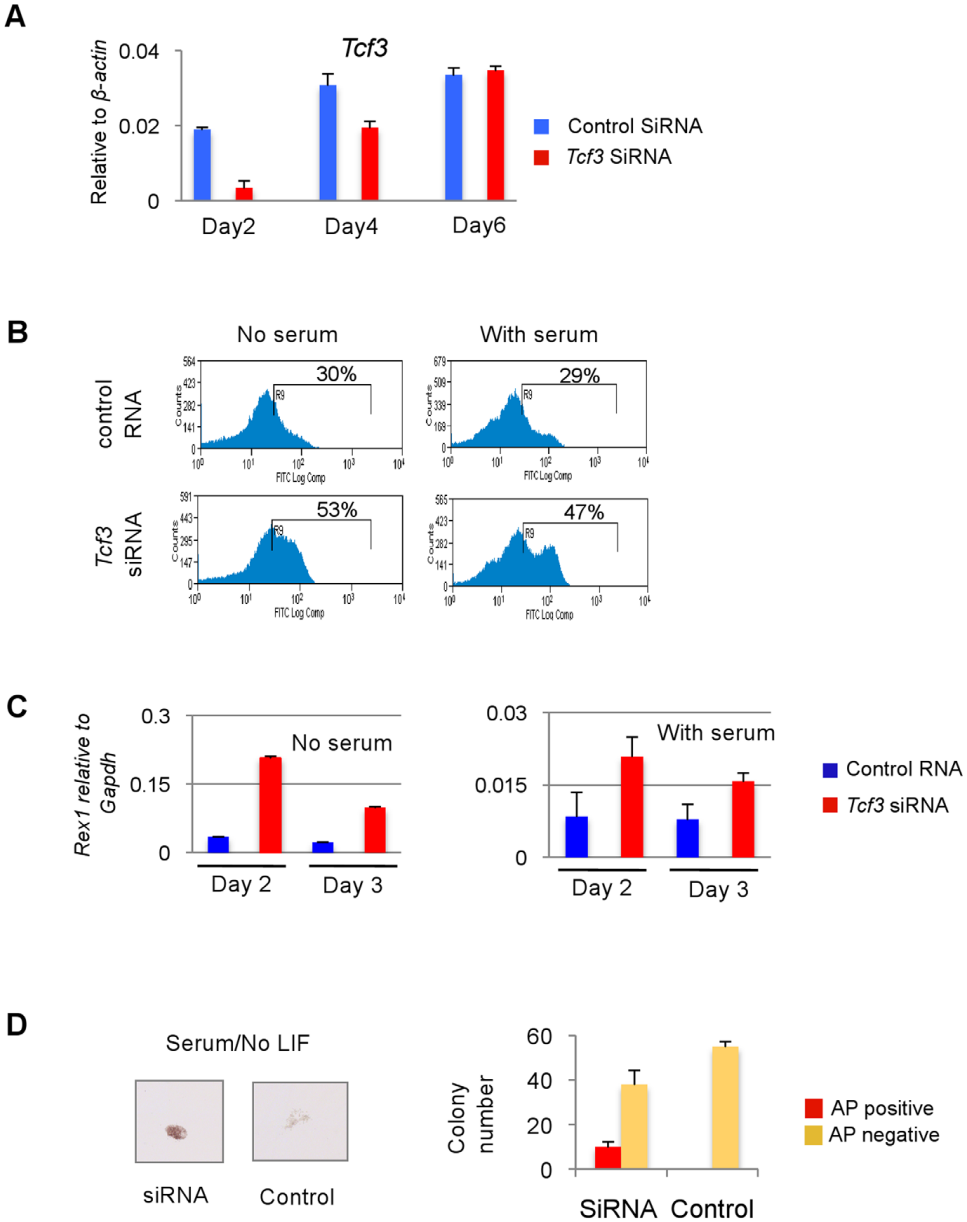
siRNA knockdown of Tcf3 in *Blm* wild type cells. **A**. qRT-PCR analysis of *Tcf3* knockdown in *Tcf3* siRNA treated ES cells and control siRNA treated cells. **B**. Graph shows population of Rex1-EGFP positive cells in *Tcf3* siRNA and control siRNA treated cells after 2 days in monolayer differentiation with or without serum. **C**. qRT-PCR analysis of *Rex1* expression in *Tcf3* siRNA or control siRNA treated cells in monolayer differentiation with or without serum. **D**. Images showing a typical AP positive ES cell colony formed in *Tcf3* siRNA treated cells after 5 days in serum while only differentiated colonies formed from control siRNA treated cells. Error bar represents standard deviation from three individual plating.

To rule out any effect specific to the *Blm-*deficient genetic background, we used siRNA to knock down *Tcf3* in wild type *Rex1* reporter ES cells. qRT-PCR showed that *Tcf3* mRNA was reduced to less than 20% two days after *Tcf3* siRNA transfection. This effect was transient and after six days *Tcf3* mRNA was restored (Fig. 6A). In *Tcf3* siRNA treated cells Rex1 expression levels remained high in serum or serum-free differentiation conditions for 2–3 days (Fig. 6B and 6C). *Tcf3* knockdown also allowed transient clonal expansion in serum without LIF. Compact alkaline phosphatase positive undifferentiated ES cell colonies were present in siRNA treated cultures 5 days after transfection and plating, while control siRNA treated cells formed only differentiated colonies (Fig. 6D).

**Figure 7:**
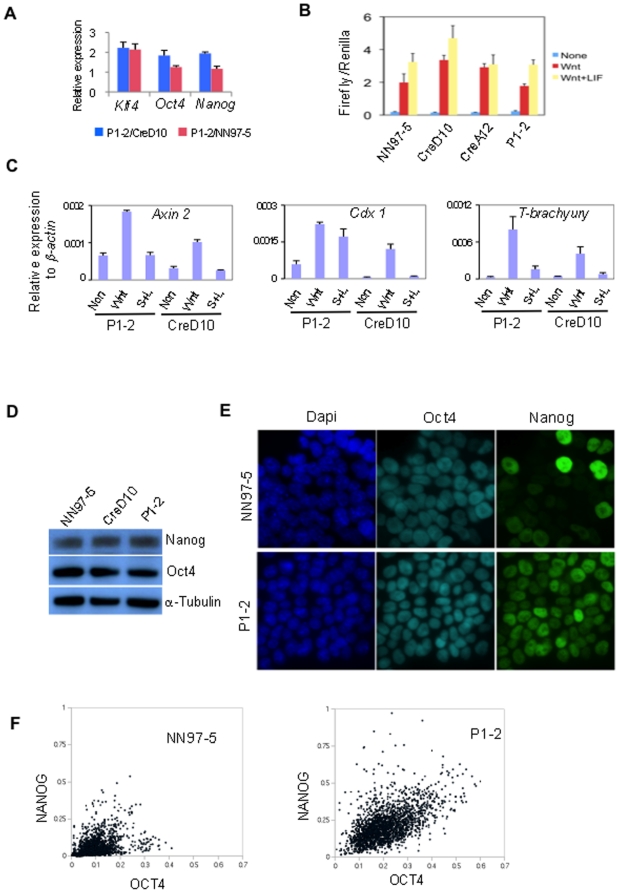
*Tcf3* mutation has subtle molecular consequences. **A**. Relative gene expression analysis by qRT-PCR in *Tcf3* mutant (P1-2) compared to Cre-reverted (CreD10)(Blue column) and wild type (NN97-5)(Red column). **B**. TOPFlash assay of Tcf-mediated transcriptional activation. None, N2B27 alone; Wnt, Wnt3A; Wnt+LIF, Wnt3A plus LIF. **C**. qRT-PCR analyses of Wnt target gene expression. S+L, Serum plus LIF. **D**. Immunoblotting analysis of Nanog and Oct4 protein expression in serum and LIF. **E**. NN97-5 and P1-2 cells cultured in serum and LIF immunostained for Oct4 and Nanog. Images show typical heterogeneous Nanog protein expression in NN97-5 cells compared to more uniform staining in P1-2 cells. **F**. Mean nuclear staining intensity of Oct4 and Nanog in individual cell was quantified using Cell profiler software and presented as a scatter plot using Microsoft Excel. 1600 cells were scored for NN97-5 and 2172 cells for P1-2. The experiment has been repeated three times.

Tcf3 is the predominant Tcf in ES cells [17]. Other Tcfs are mediators of canonical Wnt/β-catenin induced transcriptional activation, but the role of Tcf3 in this pathway is less well-defined [17]. Despite the lack of Tcf3, P1-2 cells retained TOPFlash reporter activation in response to Wnt3a (Fig. 7B). Furthermore they showed induction of chromosomal Wnt target genes, *Axin2*, *Cdx1* and *T-brachyury* (Fig. 7C). Absence of Tcf3 therefore does not impede canonical Wnt signalling in ES cells.

Genome location analyses suggest that Tcf3 binds to promoters of several pluripotency genes including *Oct4*, *Nanog*, and *Klf4* [18], [19]. Through interaction with Groucho family members Tcf3 is proposed to repress pluripotent gene expression [17]. We detected near two folds increase in the expression of the core pluripotency genes, *Oct4*, *Klf4* and *Nanog* in P1-2 cells when compared to the reverted CreD10 cells. However, when compared with NN97-5 cells only *Klf4* showed significantly increased expression (Fig. 7A). This biological variation between parental line and subclone indicates that the repressive effect of Tcf3 on individual genes may be modest and environmental factors. Nonetheless, the increased expressions of Klf4 or Nanog are notable because either of these is sufficient to increase resistance to differentiation [20], [21], [22], [23].

Western-blotting analysis indicated that neither Oct4 nor Nanog protein are appreciably increased in *Tcf3* deficient cells (Fig. 7D). We therefore examined cellular expression by immunofluorescent staining because Nanog is heterogeneous in ES cells in serum [24]. This dynamic heterogeneity is postulated to underlie ES cell susceptibility to differentiation [24], [25], [26]. Compared with NN97-5 cells, P1-2 cells cultured in serum with LIF showed more uniform immunofluorescent staining for Nanog (Fig. 7E). We quantified staining intensity relative to Oct4 over 25 fields using CellProfiler software [27]. Scatter plots of mean fluorescence intensities confirm that the fraction of low or non-expressing cells within the Oct4 positive population is reduced in *Tcf3* deficient cells (Fig. 7F). Thus absence of Tcf3 stabilises expression of Nanog within individual ES cells, even though overall expression level may not be significantly altered. Interestingly there was also a modest shift in the Oct4 profile towards higher expression, consistent with evidence that Tcf3 may repress Oct4 [18].

## Discussion

In this study, we piloted a recessive screening strategy to identify genes modulating ES cell differentiation and self-renewal. There are three key components in this approach. First, use of PB transposon mutagens offers significant advantages for genome-wide screens. They have much higher chromosomal integration efficiencies than plasmids and do not appear to have the bias for hot spots seen with retroviral vectors [4], [5]. Second, rapid and stringent selection is critical in an ES cell self-renewal screen to minimise paracrine interactions between residual undifferentiated ES cells and differentiating progeny [20]. Oct4 is widely used as a reporter and selection driver, but it is not optimal because expression reduces only gradually. Moreover, in early derivatives of ES cells, including stable EpiSC cell lines, Oct4 is fully maintained [28]. Indeed we found that selection for Oct4 was of limited utility over the time course of monolayer differentiation, with high background necessitating multiple rounds of replating. In contrast *Rex1* selection allowed mutants to be isolated after only a single round of secondary plating. Third, it is essential to demonstrate reversion of phenotype in order to confirm causality. Using the PB vector reversion can readily be achieved by excision of the gene trap cassette with Cre recombinase.

From 2,000 gene traps, we isolated ES cells with enhanced self-renewal. All 5 non-differentiating clones had a gene trap insertion disrupting the *Tcf3* gene and no Tcf3 mRNA was detectable in these cells. The integration site was identical in these clones indicating that they arose from the same original PB insertion. Some colonies exhibited partially differentiation-resistant phenotypes and also contained this Tcf3 insertion. The presence of Tcf3 mRNA in these cells indicates either that they have not converted to homozygosity or that they are mixed clones. Complete *Tcf3* deficiency greatly reduced differentiation and allowed ES cell expansion without exogenous LIF, even at clonal density. These findings are consistent with recent studies linking Tcf3 to the core pluripotent transcription factor network [17], [18]. Isolation from a stringent genetic screen independently establishes the importance of Tcf3 for ES cell differentiation. The more homogenous expression of Nanog in *Tcf3* mutants indicates that repression by Tcf3 contributes significantly to the heterogeneous and fluctuating pattern observed in serum [24], [26]. This effect is rather subtle in terms of quantitative gene expression at the population level, but is likely to be biologically significant at the single cell level. With Tcf3 deleted, Nanog is maintained more evenly in all cells and the population is therefore more resistant to inductive cues for commitment. In a separate study we present evidence that the potent impact of glycogen synthase kinase-3 inhibition on ES cell self-renewal is in large part mediated by Tcf3 derepression [29]. Genome location studies suggest that Tcf3 may directly repress multiple components of the pluripotent circuitry [18], [19]. We hypothesise that the strong phenotype of *Tcf3* deletion reflects cumulative impact of general derepression of the pluripotency network rather than dramatic up-regulation of specific targets.

In summary, this study demonstrates the feasibility of recessive genetic screening for pluripotency regulators using a PB-based gene trap in *Blm*-deficient ES cells configured for *Rex1* selection. This screen could readily be scaled up and applied in different culture conditions. Ideally, ES cells with inducible deletion of *Blm* would be used to minimise the incidence of background mutations [30]. Importantly, revertible insertional mutagenesis is a more robust screening methodology than RNAi based approaches, which although flexible inevitably suffer from variable penetrance and off-target effects.

## Methods

### ES cell culture and differentiation

Mouse ES cells were routinely maintained on gelatin coated tissue culture plates in medium containing serum and LIF as described [31]. The monolayer neural differentiation protocol is detailed in full elsewhere [8]. In brief, cells were dissociated with trypsin and washed once in PBS to remove residual FCS, and then plated in N2B27 medium at a density of 2×10^4^ cells/cm^2^. Medium was changed every second day. For non-neural differentiation, cells were plated at similar density with either recombinant BMP-4 (10 ng/ml, R&D systems) or 10% FCS. For colony assays 600 fully dissociated ES cells were plated per 90 mm tissue culture plate. Colonies were stained for alkaline phosphatase (Sigma Aldrich, cat number 86R1KT). Colonies were scored based on alkaline phosphase staining as pure ES cells, mixed or completely differentiated.

### *Rex1* knock-in

The *Rex1* coding region in AB2.2 BAC clone (bMQ-381F12, provided by Wellcome Trust Sanger Institute), was first replaced with *eGFPIrespuro* using bacterial recombineering [32]. To generate the *Rex1* targeting vector the 5′ homology arm and the 3′ homology arms including the *eGFPIrespuro* cassette were amplified by PCR and cloned into pBluescript by three-way ligation. The targeting vector was transfected into *Blm* mutant or E14Tg2aIVC ES cells by electroporation. Following 7 days puromycin (1 µM) selection ES cell colonies were picked and expanded. Genomic PCR was used to identify targeted clones. RT-PCR confirmed that only targeted clones expressed the fused transcript including first exon of *Rex 1* and the *eGFP-IresPuro* knock-in cassette.

### *PB* gene trap system

*PB 5′TR* and *PB 3′TR* with LoxP sites were amplified by PCR from plasmid *PB-SB-PGK-Neo-bPA* [4] and ligated to pBluescript to generate *pGG81*. An oligo linker was inserted to *pGG81* to generate pGG83 containing multiple cloning sites. The *SAIRESβgeo* cassette was generated by four-way ligation of *IRES* fragment from *pCA1* [33], the PCR amplified splice acceptor (*SA*) fragment and the *LacZ/Neo/bPA* fragment from RGTV-1 [2] into pBlueScript. *SAIRESβgeo* was then inserted to the pGG83 to generate the PB based gene trap vector, *pGG85.* Splinkerette PCR was performed as described [34]. In brief, genomic DNA was digested with *BstYI* and then ligated with Splinkerette oligo adapter. The genome and PB insertion junction was amplified with HMSP-1/PB-SP1 primers and then nested PCR using HMSP-2/PB-SP2 primers. PCR reaction was treated with Exonuclease I (New England Biolabs) to degrade single strand oligonucleotides, followed by ethanol precipitation for sequencing with SP3 primers.

### Luciferase assay

Cells were co-transfected with TOPFlash and Renilla plasmids using Lipofectamine^TM^ 2000 (Invitrogen). Luciferase assay was performed using Dual Luciferase Reporter Assay System (Promega). Recombinant mouse Wnt-3A was purchased from R&D Systems.

### siRNA knock down

Tcf3 siRNA (ON-TARGETplus SMARTpool L-04861-01-0005) and the control siRNA (ON-TARGETplus Non-targeting pool D-001810-10-05) were purchased from Dharmacon. 10 nM siRNA or control was used for each transfection with Lipofectamine^TM^ RNAiMAX (Invitrogen).

### Quantitative RT-PCR

Total RNA was prepared using RNeasy mini Kit (Qiagen). First strand cDNA was synthesised using Superscript^TM^ III reverse transcriptase (Invitrogen) and Oligo-dT priming. Real time PCR was performed using Taqman probes (Applied Biosystems) or the universal probe library (Roche). Relative expression was determined using the delta Ct method. Standard deviation was calculated on three PCR triplicates.

### Flow cytometry analysis

For live cell analysis, ES cells were collected in PBS with 3% FCS. ToPro-3 (Invitrogen) was added to cells at a final concentration of 0.05 nM for staining of dead cells. Analyses were performed using a CyAn flow cytometer (DakoCytomation).

### Immunoflurescence

Cells were fixed with 4% PFA at room temperature for 15 minutes and then permeabilised with PBST (0.3% Triton x-100 in PBS). Cells were then blocked and antibody stained in PBST containing 3% donkey serum. For Nanog mosaic expression analysis, 5000 cells were seeded on gelatin coated glass slides and cultured for three days to form small cell patches for antibody staining. Random fields were imaged under constant conditions using a DMI4000B microscope (Leica micosystems) using a 60× objective. Images were analysed using Cell Profiler [27] to identify DAPI labelled nuclei by Otsu thresholding, and measure the intensity of OCT4 and NANOG immunolabelling in the detected areas. Data are presented as a scatter plot of OCT4 vs NANOG intensities. Oct4 antibody is from Santa Cruz Biotechnology (sc-5249, 1∶200) and Nanog antibody is from eBioscience (14-5761-80, 1∶200). Secondary antibody for OCT4 in this assay is goat anti-mouse IgG Alex 647 and secondary for Nanog is goat anti-rat IgG Alex 488.

PCR primers and qPCR probe details are provided in supplementary information.

## References

[1] Luo G, Santoro IM, McDaniel LD, Nishijima I, Mills M (2000). Cancer predisposition caused by elevated mitotic recombination in Bloom mice.. Nat Genet.

[2] Guo G, Wang W, Bradley A (2004). Mismatch repair genes identified using genetic screens in Blm-deficient embryonic stem cells.. Nature.

[3] Ding S, Wu X, Li G, Han M, Zhuang Y (2005). Efficient transposition of the piggyBac (PB) transposon in mammalian cells and mice.. Cell.

[4] Wang W, Lin C, Lu D, Ning Z, Cox T (2008). Chromosomal transposition of PiggyBac in mouse embryonic stem cells.. Proc Natl Acad Sci U S A.

[5] Wang W, Bradley A, Huang Y (2009). A piggyBac transposon-based genome-wide library of insertionally mutated Blm-deficient murine ES cells.. Genome Res.

[6] Smith AG, Heath JK, Donaldson DD, Wong GG, Moreau J (1988). Inhibition of pluripotential embryonic stem cell differentiation by purified polypeptides.. Nature.

[7] Williams RL, Hilton DJ, Pease S, Willson TA, Stewart CL (1988). Myeloid leukaemia inhibitory factor maintains the developmental potential of embryonic stem cells.. Nature.

[8] Kunath T, Saba-El-Leil MK, Almousailleakh M, Wray J, Meloche S (2007). FGF stimulation of the Erk1/2 signalling cascade triggers transition of pluripotent embryonic stem cells from self-renewal to lineage commitment.. Development.

[9] Lowell S, Benchoua A, Heavey B, Smith AG (2006). Notch promotes neural lineage entry by pluripotent embryonic stem cells.. PLoS Biol.

[10] Smith AG (2001). Embryo-derived stem cells: of mice and men.. Annu Rev Cell Dev Biol.

[11] Toyooka Y, Shimosato D, Murakami K, Takahashi K, Niwa H (2008). Identification and characterization of subpopulations in undifferentiated ES cell culture.. Development.

[12] Wray J, Kalkan T, Smith AG (2010). The ground state of pluripotency.. Biochem Soc Trans.

[13] Mountford PS, Smith AG (1995). Internal ribosome entry sites and dicistronic RNAs in mammalian transgenesis.. Trends Genet.

[14] Cary LC, Goebel M, Corsaro BG, Wang HG, Rosen E (1989). Transposon mutagenesis of baculoviruses: analysis of Trichoplusia ni transposon IFP2 insertions within the FP-locus of nuclear polyhedrosis viruses.. Virology.

[15] Devon RS, Porteous DJ, Brookes AJ (1995). Splinkerettes—improved vectorettes for greater efficiency in PCR walking.. Nucleic Acids Res.

[16] Ying QL, Stavridis M, Griffiths D, Li M, Smith A (2003). Conversion of embryonic stem cells into neuroectodermal precursors in adherent monoculture.. Nat Biotechnol.

[17] Pereira L, Yi F, Merrill BJ (2006). Repression of Nanog gene transcription by Tcf3 limits embryonic stem cell self-renewal.. Mol Cell Biol.

[18] Cole MF, Johnstone SE, Newman JJ, Kagey MH, Young RA (2008). Tcf3 is an integral component of the core regulatory circuitry of embryonic stem cells.. Genes Dev.

[19] Tam WL, Lim CY, Han J, Zhang J, Ang YS (2008). Tcf3 Regulates Embryonic Stem Cell Pluripotency and Self-Renewal by the Transcriptional Control of Multiple Lineage Pathways..

[20] Chambers I, Colby D, Robertson M, Nichols J, Lee S (2003). Functional expression cloning of Nanog, a pluripotency sustaining factor in embryonic stem cells.. Cell.

[21] Li Y, McClintick J, Zhong L, Edenberg HJ, Yoder MC (2005). Murine embryonic stem cell differentiation is promoted by SOCS-3 and inhibited by the zinc finger transcription factor Klf4.. Blood.

[22] Hall J, Guo G, Wray J, Eyres I, Nichols J (2009). Oct4 and LIF/Stat3 additively induce Kruppel factors to sustain embryonic stem cell self-renewal.. Cell Stem Cell.

[23] Niwa H, Ogawa K, Shimosato D, Adachi K (2009). A parallel circuit of LIF signalling pathways maintains pluripotency of mouse ES cells.. Nature.

[24] Chambers I, Silva J, Colby D, Nichols J, Nijmeijer B (2007). Nanog safeguards pluripotency and mediates germline development.. Nature.

[25] Silva J, Smith A (2008). Capturing pluripotency.. Cell.

[26] Kalmar T, Lim C, Hayward P, Munoz-Descalzo S, Nichols J (2009). Regulated fluctuations in nanog expression mediate cell fate decisions in embryonic stem cells.. PLoS Biol.

[27] Carpenter AE, Jones TR, Lamprecht MR, Clarke C, Kang IH (2006). CellProfiler: image analysis software for identifying and quantifying cell phenotypes.. Genome Biol.

[28] Rathjen J, Lake JA, Bettess MD, Washington JM, Chapman G (1999). Formation of a primitive ectoderm like cell population, EPL cells, from ES cells in response to biologically derived factors.. J Cell Sci.

[29] Wray J, Kalkan T, Gomez-Lopez S, Eckardt D, Kemler R (2011). Inhibition of glycogen synthase kinase-3 alleviates Tcf3 repression of the pluripotency network and increases embryonic stem cell resistance to differentiation..

[30] Yusa K, Horie K, Kondoh G, Kouno M, Maeda Y (2004). Genome-wide phenotype analysis in ES cells by regulated disruption of Bloom's syndrome gene.. Nature.

[31] Smith AG (1991). Culture and differentiation of embryonic stem cells.. J Tiss Cult Meth.

[32] Liu P, Jenkins NA, Copeland NG (2003). A highly efficient recombineering-based method for generating conditional knockout mutations.. Genome Res.

[33] Abram CL, Page MJ, Edwards PA (1997). A new retroviral vector, CA1, to identify and select for cells expressing an inserted gene in vitro and in vivo.. Gene.

[34] Mikkers H, Allen J, Knipscheer P, Romeijn L, Hart A (2002). High-throughput retroviral tagging to identify components of specific signaling pathways in cancer.. Nat Genet.

